# *Taenia solium* Cysticercosis in the Democratic Republic of Congo: How Does Pork Trade Affect the Transmission of the Parasite?

**DOI:** 10.1371/journal.pntd.0000817

**Published:** 2010-09-07

**Authors:** Nicolas Praet, Kirezi Kanobana, Constantin Kabwe, Vivi Maketa, Philippe Lukanu, Pascal Lutumba, Katja Polman, Peter Matondo, Niko Speybroeck, Pierre Dorny, Julienne Sumbu

**Affiliations:** 1 Institute of Tropical Medicine, Antwerp, Belgium; 2 Department of Infectious and Parasitic Diseases, Research Unit of Epidemiology and Risk Analysis Applied to Veterinary Sciences, Faculty of Veterinary Medicine, University of Liege, Liege, Belgium; 3 Institut National de Recherche Biomédicale, Kinshasa, Democratic Republic of Congo; 4 Tropical Medicine Department, Kinshasa University, Kinshasa, Democratic Republic of Congo; 5 Kimpese Health Zone, Kimpese, Democratic Republic of Congo; 6 Laboratoire Vétérinaire de Kinshasa, Kinshasa, Democratic Republic of Congo; 7 Institute of Health and Society, Université Catholique de Louvain, Brussels, Belgium; Universidad Peruana Cayetano Heredia, Peru

## Abstract

**Background:**

*Taenia solium*, a zoonotic parasite that is endemic in most developing countries where pork is consumed, is recognised as the main cause of acquired epilepsy in these regions. *T. solium* has been reported in almost all of the neighboring countries of Democratic Republic of Congo (DRC) but data on the current prevalence of the disease in the country itself are lacking. This study, focusing on porcine cysticercosis (CC), makes part of a first initiative to assess whether cysticercosis is indeed actually present in DRC.

**Methods:**

An epidemiological study on porcine CC was conducted (1) on urban markets of Kinshasa where pork is sold and (2) in villages in Bas-Congo province where pigs are traditionally reared. Tongue inspection and ELISA for the detection of circulating antigen of the larval stage of *T. solium* were used to assess the prevalence of active CC in both study sites.

**Findings:**

The overall prevalence of pigs with active cysticercosis did not significantly differ between the market and the village study sites (38.8 [CI95%: 34–43] versus 41.2% [CI95%: 33–49], respectively). However, tongue cysticercosis was only found in the village study site together with a significantly higher intensity of infection (detected by ELISA).

**Interpretation:**

Pigs reared at village level are sold for consumption on Kinshasa markets, but it seems that highly infected animals are excluded at a certain level in the pig trade chain. Indeed, preliminary informal surveys on common practices conducted in parallel revealed that pig farmers and/or buyers select the low infected animals and exclude those who are positive by tongue inspection at village level. This study provides the only recent evidence of CC presence in DRC and gives the first estimates to fill an important gap on the African taeniasis/cysticercosis distribution map.

## Author Summary

*Taenia solium* is a parasite that can affect both humans and pigs, causing important economic losses in pig production and being the main cause of acquired epilepsy in endemic areas. However, the parasite has been neglected in many African countries and particularly in the Democratic Republic of Congo (DRC), where recent data are non-existent. The present study is part of a first initiative to assess whether cysticercosis is actually present in DRC and to estimate its potential economic and public health importance. Focusing our work on porcine cysticercosis, we demonstrated high prevalence figures of active infections in villages in a rural area of DRC and in markets in the city of Kinshasa. Moreover, the intensity of infection was higher in pigs sampled in villages as compared to pigs sampled on urban markets. Preliminary surveys conducted in parallel in both study sites suggest an effect of pork trade on the transmission of the parasite selecting highly infected pigs at village level.

## Introduction

*Taenia solium* taeniasis/cysticercosis is a zoonotic disease with serious public health and agricultural consequences, which is endemic in most developing countries where pork is consumed [1]. The adult tapeworm occurs only in humans (taeniasis) but infection with the larval stage (cysticercosis (CC)) can affect both pigs and humans.

Taeniasis has only mild clinical manifestations and may go unnoticed. Human cysticercosis occurs when cystic larvae lodge in muscles, subcutaneous tissues, eyes or brain. Localization in the brain or in the spinal cord causes neurocysticercosis (NCC) [2]. Seizures are the most common symptom of NCC and NCC has been reported to be the main cause of acquired epilepsy in developing countries [3], [4]. Porcine cysticercosis, equally caused by the establishment of larvae in tissues, is an economical important disease, because of condemnation of carcasses and/or reduction of meat price of infected pigs [5], [6]. The life cycle of the disease is sustained in regions with low hygienic standards, lack of sanitary conditions and traditional pig-production systems with free roaming pigs, thereby facilitating pig's access to contaminated feces from tapeworm carriers.

Taeniasis/cysticercosis is a poverty-related disease [7]. It has been seriously neglected due to the lack of information and awareness of the extent of the problem in many countries, combined with the absence of suitable and sensitive diagnostic tools which can be applied at low cost and large scale in disease-endemic areas [8], [9]. This so far neglected situation issues also in part from the fact that CC has no overt disease-specific manifestations, neither in pigs nor in humans, which makes it difficult to sensitize responsible authorities, both in the veterinary and the medical sectors. However, recently, the World Health Organization included cysticercosis in its 2008–2015 strategic plans for the control of neglected tropical diseases NTDs.

The Democratic Republic of Congo (DRC) is one of the largest, and also poorest countries of Sub Saharan Africa (SSA) with a prevalence of undernourishment of 76% as compared to 30% for SSA [10]. Moreover, the economy of the DRC is only slowly recovering from two decades of decline caused by conflicts and war. This war situation dramatically reduced national output and government revenue, increased external debt, and resulted in the deaths of more than 3.5 million people from violence, famine, and disease and consequently also reduced the government's priorities on public health [11].

It is evident that health priorities in resource poor countries focus on major diseases such as HIV/AIDS, malaria or tuberculosis. However, this does not preclude the need of addressing neglected diseases, for which relatively simple control and prevention measures are often available, but only effective if applied in an integrated and sustainable way [12].

Common NTD's such as, soil-transmitted helminth infections (STH), schistosomiasis, filariasis and onchocerciasis are known to be widespread among the poor in SSA, including the DRC [13]. NCC and CC have been reported in almost all of the neighboring countries of DRC [14], [15], but data on the current prevalence of the disease in the country itself are lacking. The last reports on human and porcine CC in DRC originate from 1958 [16] and 1990 [17], respectively (reviewed in [18]). In 2004, a report of preliminary abattoir surveys revealed the presence of cysticercosis in approximately 3% of the pigs presented at slaughter (Sumbu J., proceedings of the 3ème congrès de pathologies infectieuses et parasitiare, 10–12 Decembre 2004, Kinshasa, abstract N° F12).

Based hereon, recent incentives have emerged both from the political and scientific sectors, to catch up for these decades of negligence. The data reported here focus on porcine cysticercosis and are part of a first initiative to assess whether cysticercosis is indeed actually present in DRC and to estimate its potential economical and public health consequences.

## Materials and Methods

### Ethical clearance

The study protocol was approved by Laboratoire Vétérinaire de Kinshasa (LABOVET), national veterinary reference laboratory of The Democratic Republic of Congo. The study permissions were obtained from LABOVET, from the village leaders and from the pig owners. Lingual examination and blood sampling on pigs were conducted by professional veterinarians, according to Congolese guidelines for animal husbandry. The protocol for the village survey was approved by the Ethical Committee of the University of Kinshasa, Democratic Republic of Congo, the Institutional Review Board of the Institute of Tropical Medicine of Antwerp, Belgium, and by the Ethical Committee of the University Teaching Hospital of Antwerp, Belgium. Written informed consent was obtained from all pig owners.

### Study sites

An epidemiological study on porcine cysticercosis was conducted (1) on urban markets of Kinshasa where pork is sold and (2) in villages in Bas-Congo province where pigs are traditionally reared and consumed and where risk factors for the disease are ubiquitous.

**Figure 1:**
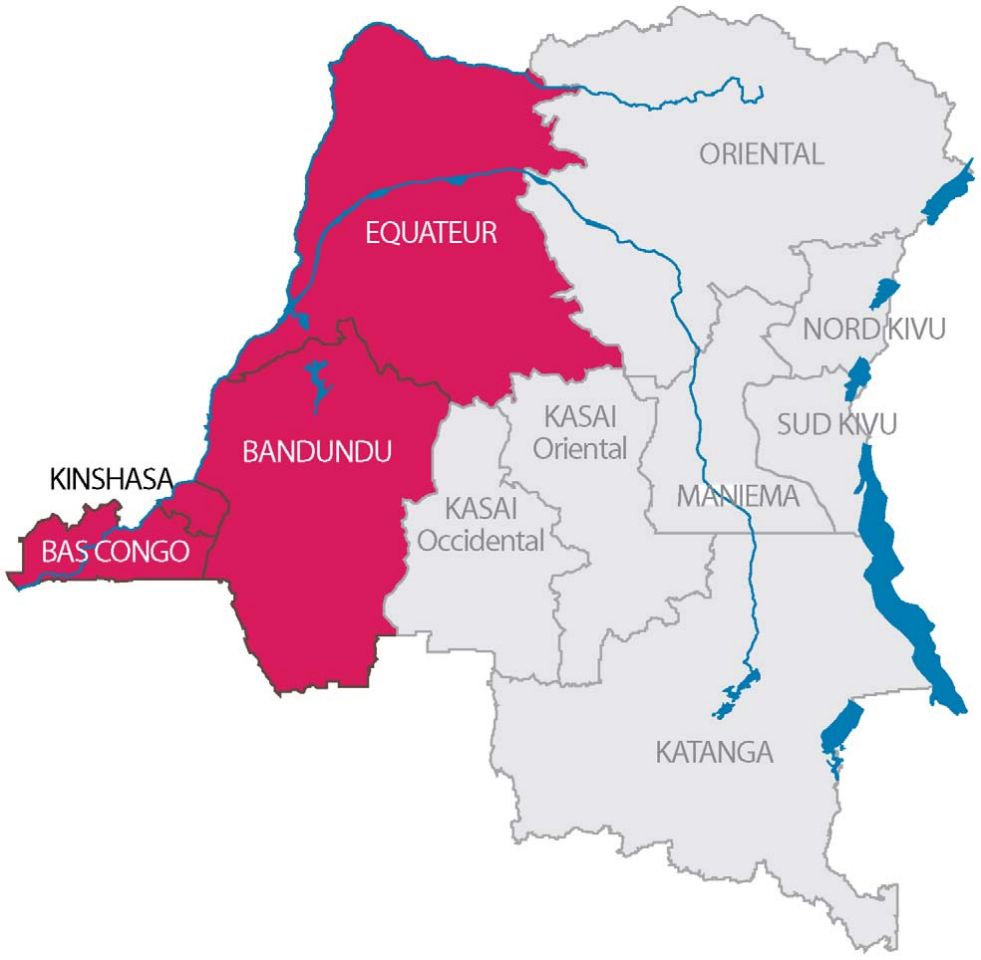
Origins of the pigs included in the market study.

The first study was conducted between November and December 2008 in 5 important markets across the city of Kinshasa. All markets were visited approximately 3 times a week in the study period. The selection of the markets was driven by the objective to target all major import routes of pigs from outside into the city, and included the following markets: Liberté, Zikida, Gambela, Matete and Grand Marché de Kinshasa (Figure 1). All pigs presented for slaughtering during the visits of the markets were included in the study.

The second study was conducted in April 2009 in 5 villages (Viaza, Kiandu, Malanga, Kimaku and Kiasungua), in the health zone of Kimpese and located within a distance radius of 30 km around Kimpese city. Agriculture represents the most important income in the area. The villages were selected based on (1) the presence of the major risk factors for transmission of *T. solium* (lack of latrines, free-roaming pigs and poor hygienic conditions) and (2) the willingness of the community to participate in the study. All pig owners were asked to participate and to present all their pigs for CC diagnosis.

All villages shared cultural, commercial, social and economical characteristics. Water supply system depends on the proximity of a river and water is non potable. Roads are not paved and there is no electricity. Inhabitants have access to primary health care through health centres, either located in their own village, or in a neighboring village. The pig phenotypes in the villages were very diverse and likely descend from mixed races with the major phenotypes of Pietrain or Landrace. Pigs were kept roaming during the day, and enclosed at night.

Pig demand and consumption may differ throughout the year. As we currently do not have evidence to support the latter, a comparable trade off was assumed for the period of November-December 2008 (market study) and April 2009 (village study).

### Diagnosis of active cysticercosis

Tongue inspection (TI) and blood sampling were performed in both surveys. TI was performed while the mouth was opened using a wooden rod, the examiner, using a cloth, gently pulled the tongue, examined and palpated it throughout the base. The pig was considered positive for cysticercosis if cyst-like nodules were either seen or felt [19]. Blood samples of approximately 10 ml were collected in dry tubes, allowed to clot at ambient temperature, centrifuged, and serum was collected. Serum was stored at −20°C until analysis and later tested with the ELISA for the detection of circulating antigen of the larval stage of *T. solium* (antigen ELISA, [20]), which enables the detection of active infections (presence of viable cysts).

The optical density of each sample was divided by the cut off value in order to obtain standardized ratios. These ratio values allow determining the positivity of each pig (ratio>1) and also reflecting the intensity of infection [21]. To illustrate the comparison of infection intensity between both surveys, 4 classes of antigen ratio (above 1, infected animals only) were arbitrarily defined: 1≤ratio<2 (low intensity), 2≤ratio<5 (medium intensity), 5≤ratio<10 (high intensity), ratio ≥10 (very high intensity).

### Statistical analysis

The data were analyzed in STATA 11. Prevalence data and their 95% confidence intervals were calculated by dividing the number of positive pigs (TI or ELISA) by the total number of pigs. Multivariate logistic regression was applied to assess whether the risk of being infected depended on the market or province of origin (market study) or on the village of origin (village study). A negative binomial-logit hurdle regression model [22] was used to compare the proportions and infection intensity of antigen ELISA positive pigs in both market and village study sites. The p value threshold for significance was set at 0.05 for all statistical analyses.

## Results

In the first study, a total of 498 pigs from the Kinshasa markets were blood sampled. Pigs originated from different types of pig-breeding systems, i.e. traditional (free-roaming and scavenging pigs) and industrial (confined pigs). In 133 of these pigs the tongue palpation was either not performed or not conclusive. Tongue cysticercosis was not detected in any of the remaining 364 pigs.

**Figure 2:**
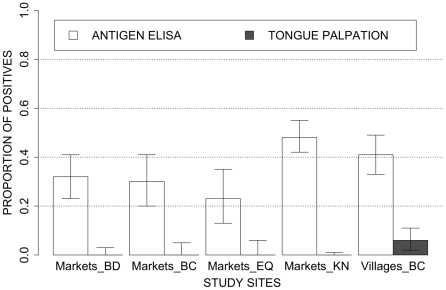
Proportion of pigs with active cysticercosis as determined by antigen ELISA (white bars) and tongue inspection (grey bars). The proportions are presented per province for the pigs sold on the markets and as overall prevalence measured in the villages (BD  =  Bandundu, BC  =  Bas-Congo, EQ  =  Equateur and KN  =  Kinshasa). Upper and lower exact 95% binomial confidence intervals are represented through error bars.

The overall prevalence of active cysticercosis (as measured by antigen ELISA) was 38.4% (CI95%: 34–43) with no significant differences in percentages of positives between the respective markets. The proportion of pigs with circulating *T. solium* antigen was significantly higher in pigs originating from the province of Kinshasa as compared to the other provinces (p value <0.01; Figure 2).

In the second study, a total of 153 pigs from the Bas-Congo villages were blood sampled. A conclusive tongue inspection was performed in 145 pigs. Tongue cysticercosis was detected among 5.5% (CI95%: 2.4–10.6). The prevalence of active cysticercosis by antigen ELISA measured among the free roaming pigs of the villages was 41.2% (CI95%: 33–49). No significant difference in percentage of positives was observed between the 5 villages (Figure 2).

**Figure 3:**
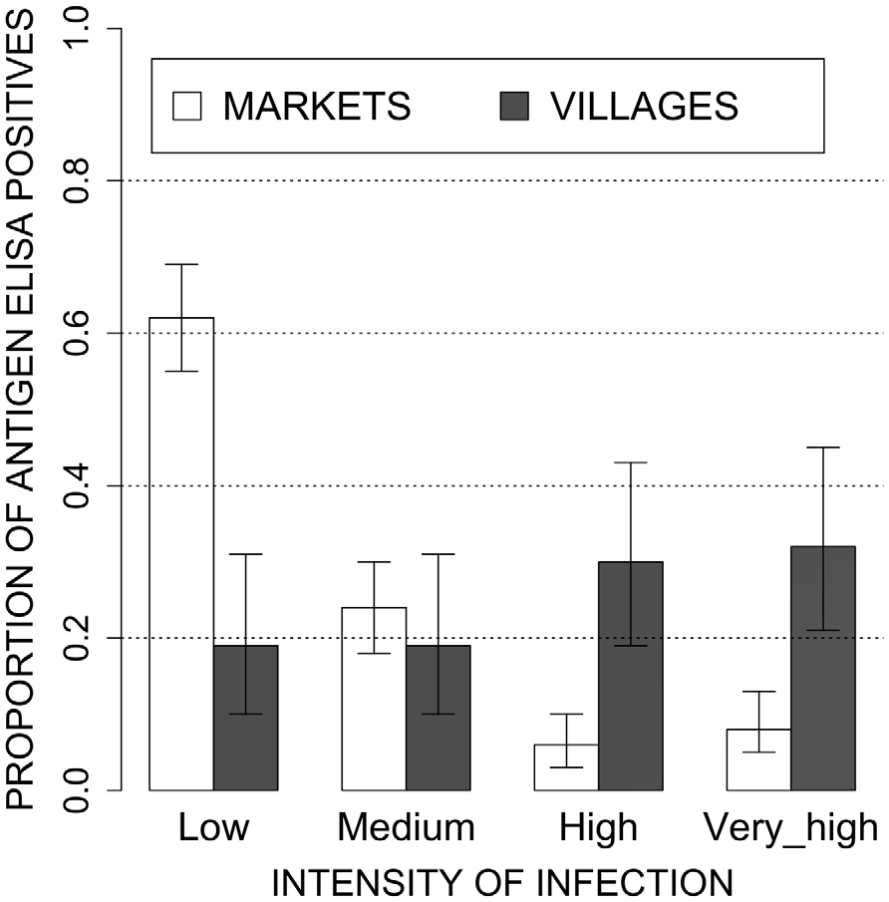
Proportion of infected pigs in function of their intensity of infection as reflected by the value of the ratio resulting from the ELISA for detection of circulating antigens. Four classes have been arbitrarily defined: 1≤ratio<2 (low intensity), 2≤ratio<5 (medium intensity), 5≤ratio<10 (high intensity), ratio ≥10 (very high intensity).

The overall prevalence of pigs with active cysticercosis as measured by the detection of circulating *T. solium* antigen did not significantly differ between the market and the village study sites (38.8 versus 41.2% respectively; p = 0.561). However, tongue cysticercosis was only found in the village study site together with a significantly higher intensity of infection as reflected by the higher proportion of infected pigs having a high or a very high ratio value in the villages (p<0.001; Figure 3).

## Discussion

Twenty years ago the last official report on the presence of porcine CC in DRC was issued [17]. Despite the high prevalence figures reported at that time (between 10 and 30%), no further research has so far been conducted on the disease within the country, neither in pigs nor in humans. Based on the knowledge that *T. solium* CC causes major economical losses and a considerable public health burden in countries surrounding DRC, joined with the presence of similar socio-economical and environmental conditions, the disease was anticipated to be equally important in DRC.

The current study is a first attempt to estimate the importance of this zoonosis in DRC, thereby focusing on the veterinary part, i.e. porcine CC, which may represent a major constraint to increase pig production in developing countries, especially affecting the rural poor. Our data demonstrate that the disease indeed still prevails in the country with apparent prevalence figures of active CC in pigs above 25% in provinces surrounding the Kinshasa Region.

The genus-specific ELISA for the detection of *T. solium* circulating antigen may show cross-reactions in pigs infected with *T. hydatigena.* The presence of this parasite in pigs in DRC needs to be further evaluated. However, veterinary carcass inspection was performed in 356 out of the 547 pigs included in the market study and *T. hydatigena* was never observed. In addition, based on abattoir surveys performed in Ituri [17] and on observations that *T. hydatigena* infections in pigs in African countries appear to be scarce [20], [23], we assume a low interference with the obtained serological results.

In order to have a more improved picture of the occurrence of porcine CC, we estimated the prevalence at two different levels of the pig trade chain: (1) in rural villages, at farming level, representing the source of infection sites and (2) in Kinshasa, at urban markets level, representing the site where veterinary inspection should take place, and prevalence of porcine CC are thus expected to be lower.

Interestingly, the mean proportion of active infections detected by antigen sero-detection did not significantly differ between both study sites, but the intensity of infection was significantly higher in pigs sampled in the villages as compared to those in the markets which was evidenced by a higher proportion of animals with antigen ratio's above 5. It seems that highly infected animals are excluded at a certain level in the pig trade chain.

Indeed, preliminary informal surveys on common practices in pig husbandry and pig trading were conducted in parallel in the two study sites. These revealed that the majority of both farmers in the villages and pig traders on the markets knew how to detect the parasite using tongue and/or carcass inspections, as it has been previously reported in other endemic regions [23], [24], [25]. Moreover, they pointed out that pig farmers and/or buyers select the low infected animals and exclude those who are positive by tongue inspection at village level. Based on their observations in slaughterhouses Ngowi et al. suggested a similar practice among pig traders in Tanzania [23].

More heavily infected pigs are subsequently used for villagers' own consumption or sold at local (clandestine) markets (data not shown).

This is in agreement with the observations of Zoli et al. [14], who notified that due to the lack of well organized meat inspection, partial or total condemnation of infected carcasses barely occurred and that a pig carcass infected with CC was sold at a decreased price.

Because of technical reasons, carcass dissection of a sub-sample of the pig populations could not be performed in these studies. However, this should be done in the future in order to confirm active infection in antigen ELISA positive pigs.

Pork sold on Kinshasa markets originated from different regions in the country and from different types of pig-breeding systems, i.e. traditional (free-roaming and scavenging pigs) and industrial (confined pigs). Since the latter would keep animals from contact with human faeces, the proportion of CC positive pigs in such conditions was expected to be low or even absent, thereby influencing the overall prevalence of the disease on the markets. However, this was not reflected by our results, which showed similar prevalence figures in the market and village study sites. The question arises whether CC is indeed absent in industrial pig farms, which deserves further investigations.

This study is the first one to provide well-documented evidence on the presence of CC in different regions of DRC. Moreover, it confirms the higher probability to find infected pigs in rural areas where pigs are traditionally reared, despite the presence of some knowledge on the disease in the rural communities. The relatively high prevalence figures for porcine CC as presented here warrant similar studies on the status of human (neuro)cysticercosis, which are currently conducted by our research group. Moreover, our work points out the importance of conducting socio-economical surveys to understand the behavior of the different stakeholders acting in the pig meat production and trade chain, from the farmer to the consumer including the middle-men. The respective studies will enable to determine the economic and public health impact of the parasite in DRC and fill an important gap on the African taeniasis/cysticercosis distribution map.
